# Supplementary material for: Sugar promotes vegetative phase change in Arabidopsis thaliana by repressing the expression of MIR156A and MIR156C
Source: eLife. 2013 Mar 26;2:e00260. doi: 10.7554/eLife.00260 (PMC3608266; doi:10.7554/eLife.00260)
Supplement: Supplementary file 1. — The genomic organization of MIR156A (At2g25095). The two most abundant transcripts are indicated. Exons are indicated in yellow. The miR156 hairpin is underlined, and the mature miRNA is indicated in blue. DOI: http://dx.doi.org/10.7554/eLife.00260.013 [file elife00260s001.doc]

Transcript 1

1 ATTCATTGTT CACTCTCAAA TCTCAAGTTC ATTGCCATTT TTAGGTCTCT

51 CTATAAATTC AAATGTTCTG TTCAATTCAA TGCGTCGCCA GACATCTGTT

101 CCCTTTGCAT GTAAGAGAGA TAAAGAAAGC GACAAGAGCC ATAAAGAAAG

151 GTAAGACTCT TTGAAATAGA GAGAGATAAG GTTTTCTCTT ATCTTCTTCT

201 CATCAGATCT TTGTTTCTTT ACCCTCTTTC TTTCTTTTTT TTGCTTTTTA

251 TGGTTATGTT TTTTCTCGAT TTAGACAAAA ACCCTAGATT TGATCTTCTA

301 AAGGGTCTCA AATGGAATCT CTTCTCTTCT CATATCTCTC CCTCTCTCCC

351 TCCCTCTCTT TGATTCTTTG TCTTCTCCAG TTAAAACTCA GATCTAACAC

401 AAAGCTTAAA AGATTCTCAT CGTTTCTTGT TTTCTTTGTT TCATCTTGTA

451 GATCTCTGAA GTTGGACTAA TTGTGAATGA AAGAGTTGGG ACAAGAGAAA

501 CGCAAAGAAA CTGACAGAAG AGAGTGAGCA CACAAAGGCA ATTTGCATAT

551 CATTGCACTT GCTTCTCTTG CGTGCTCACT GCTCTTTCTG TCAGATTCCG

601 GTGCTGATCT CTTTGGCCTG TCTTCGTTCT CTATGTCTCA ATCTCTCTCT

651 ATCACTTACA CAATCTTTCT CTGTCTCTCT TGGTTATCTC CATATTAAGG

701 TAAGAAGATA CGATCTAAAC TCTGGCTTGT TCTTGTTTCT AGATTTTTTG

751 TGCCATCTAC TTGCATGCGG GAGGAGACCT TTAGCCTTTA ATCATTCATT

801 ATATTTTAAG AGATGGGTAG TATCGAAAAA AGCTTAATCT ATTAGTTAAC

851 GCGTGGTTAA GTGAGATTAG CAATGTAACC TAGTTGTTAA TAGGCTGGTC

901 CTTAATTTTT CTCGAGTCAT GTATATATTA TATCGTTTCT TTTTCCACTC

951 TGTCTCTCTC GAGGCGTTTT AATCCCTAGT TTCCTTTGTA AACATATAAT

1001 TTATGTCTTT TGATTCATGC GATCATATAT TATCTCATTT TGCGTGAATA

1051 AGATGTTAAG GTCATGATAT TTGCATTATA AGAATGAGTC GTTGCTTTTC

1101 TTGTTATTTA AATGGTTTGC TCGTACATCT TTATTCCATG AGGTGCATGA

1151 TTAGCTTATT ATTTAACAAA ATAGGTATAT GTTAGTATAT AATTAGTTGT

1201 ACTTACATTT TTTCTACATT TTATATGTAT ATATTTGATT CCATGTTACA

1251 TTTGTGTACA TGAATATTGC GAGAATATTC TTTTAGGTGT TTTTATGGTT

1301 AGATTAACTT GAGAACATGT TCCCTGGCAA CTGCATCTCT CAAACACACA

1351 TTTTGTATGC AAAACTAATA CAGCAGCCAA TAATGTATGA GTTTGTTGAA

1401 TAGGTAGCAC AAGTTGATGT GTCATGTCTT CCCACTTTTG TACTGTTAAT

1451 ACTGTTGAAT TTTTTTGGTG TTTGTCGACA AAATATCAAT ACTACAATTA

1501 ATTTTTGTCC TACTGTTACA CCTTTTAGTT TTAACTTGGG TAAGTGAGTC

1551 GTTGGACTAA AAATCACGAT GATTATGTGG TCAAATATGT GTATCTTGGT

1601 GTATATTTGT ATTTTCTGTG ATTAAGATGA TTGCATCTTT AAGACATATT

1651 TGTAGCTTTA ATTGAAGTCA ATACTGATAT TTCTGAAGAG ATTGGGAAAT

1701 TTGGAAGACA TGGAATATTG GTGAACTTTG TTTCTGACTT TAATGATTTC

1751 AGAGTTGTTG GTTCCAGCTT TATTAGTTCG CTTCATTTCA TATATTCAAA

1801 AAAAAAAAAA AACTTTCCTT TCTCATCAAA TCTATTGATA GTTCTAAAAT

Transcript 2

1 ATTCATTGTT CACTCTCAAA TCTCAAGTTC ATTGCCATTT TTAGGTCTCT

51 CTATAAATTC AAATGTTCTG TTCAATTCAA TGCGTCGCCA GACATCTGTT

101 CCCTTTGCAT GTAAGAGAGA TAAAGAAAGC GACAAGAGCC ATAAAGAAAG

151 GTAAGACTCT TTGAAATAGA GAGAGATAAG GTTTTCTCTT ATCTTCTTCT

201 CATCAGATCT TTGTTTCTTT ACCCTCTTTC TTTCTTTTTT TTGCTTTTTA

251 TGGTTATGTT TTTTCTCGAT TTAGACAAAA ACCCTAGATT TGATCTTCTA

301 AAGGGTCTCA AATGGAATCT CTTCTCTTCT CATATCTCTC CCTCTCTCCC

351 TCCCTCTCTT TGATTCTTTG TCTTCTCCAG TTAAAACTCA GATCTAACAC

401 AAAGCTTAAA AGATTCTCAT CGTTTCTTGT TTTCTTTGTT TCATCTTGTA

451 GATCTCTGAA GTTGGACTAA TTGTGAATGA AAGAGTTGGG ACAAGAGAAA

501 CGCAAAGAAA CTGACAGAAG AGAGTGAGCA CACAAAGGCA ATTTGCATAT

551 CATTGCACTT GCTTCTCTTG CGTGCTCACT GCTCTTTCTG TCAGATTCCG

601 GTGCTGATCT CTTTGGCCTG TCTTCGTTCT CTATGTCTCA ATCTCTCTCT

651 ATCACTTACA CAATCTTTCT CTGTCTCTCT TGGTTATCTC CATATTAAGG

701 TAAGAAGATA CGATCTAAAC TCTGGCTTGT TCTTGTTTCT AGATTTTTTG

751 TGCCATCTAC TTGCATGCGG GAGGAGACCT TTAGCCTTTA ATCATTCATT

801 ATATTTTAAG AGATGGGTAG TATCGAAAAA AGCTTAATCT ATTAGTTAAC

851 GCGTGGTTAA GTGAGATTAG CAATGTAACC TAGTTGTTAA TAGGCTGGTC

901 CTTAATTTTT CTCGAGTCAT GTATATATTA TATCGTTTCT TTTTCCACTC

951 TGTCTCTCTC GAGGCGTTTT AATCCCTAGT TTCCTTTGTA AACATATAAT

1001 TTATGTCTTT TGATTCATGC GATCATATAT TATCTCATTT TGCGTGAATA

1051 AGATGTTAAG GTCATGATAT TTGCATTATA AGAATGAGTC GTTGCTTTTC

1101 TTGTTATTTA AATGGTTTGC TCGTACATCT TTATTCCATG AGGTGCATGA

1151 TTAGCTTATT ATTTAACAAA ATAGGTATAT GTTAGTATAT AATTAGTTGT

1201 ACTTACATTT TTTCTACATT TTATATGTAT ATATTTGATT CCATGTTACA

1251 TTTGTGTACA TGAATATTGC GAGAATATTC TTTTAGGTGT TTTTATGGTT

1301 AGATTAACTT GAGAACATGT TCCCTGGCAA CTGCATCTCT CAAACACACA

1351 TTTTGTATGC AAAACTAATA CAGCAGCCAA TAATGTATGA GTTTGTTGAA

1401 TAGGTAGCAC AAGTTGATGT GTCATGTCTT CCCACTTTTG TACTGTTAAT

1451 ACTGTTGAAT TTTTTTGGTG TTTGTCGACA AAATATCAAT ACTACAATTA

1501 ATTTTTGTCC TACTGTTACA CCTTTTAGTT TTAACTTGGG TAAGTGAGTC

1551 GTTGGACTAA AAATCACGAT GATTATGTGG TCAAATATGT GTATCTTGGT

1601 GTATATTTGT ATTTTCTGTG ATTAAGATGA TTGCATCTTT AAGACATATT

1651 TGTAGCTTTA ATTGAAGTCA ATACTGATAT TTCTGAAGAG ATTGGGAAAT

1701 TTGGAAGACA TGGAATATTG GTGAACTTTG TTTCTGACTT TAATGATTTC

1751 AGAGTTGTTG GTTCCAGCTT TATTAGTTCG CTTCATTTCA TATATTCAAA

1801 AAAAAAAAAA AACTTTCCTT TCTCATCAAA TCTATTGATA GTTCTAAAAT

**Supplementary file 1:** The genomic organization of MIR156A (At2g25095)

The two most abundant transcripts are indicated. Exons are indicated in yellow. The

miR156 hairpin is underlined, and the mature miRNA is indicated in blue.
