# Supplementary material for: Sugar promotes vegetative phase change in Arabidopsis thaliana by repressing the expression of MIR156A and MIR156C
Source: eLife. 2013 Mar 26;2:e00260. doi: 10.7554/eLife.00260 (PMC3608266; doi:10.7554/eLife.00260)
Supplement: Supplementary file 2. — The genomic organization of MIR156C (At4g31877). Exons are indicated in yellow. The miR156 hairpin is underlined, and the mature miRNA is indicated in blue. A variant 5′ end of the transcript is indicated in green. DOI: http://dx.doi.org/10.7554/eLife.00260.014 [file elife00260s002.doc]

1 CTCTTATTAA TCTAATCCTC CTCCCCGAAT ATTTCTCTGC CTTTAGTTCT

51 TTCTTTTTTG GTAATATATT TATTTTTCGT TACGATTTGG TCAAAACCCT

101 AGATTTGTTT TCCAAAAGCA TATCTGAAAA TGAAGGACAA CTTTCCTCTT

151 CTCCTTCGGT TATAAATATT CTCTCCGGTT TTGCTTGTTT AACCTAAAAG

201 CCTCAGATCT AACTCCAACA CCTTCAAAGT CTGCCTCCTT TCCAATCTTC

251 TTTCTTCTGT TCGATCTCTA ATCTCAGAAT TTGTGTCGGT AAGGTAAAGG

301 TGATAATGAG TGATGACTGA TGAGGGAGTT TTGGGACAAA TTTTAAGAGA

351 AACGCATAGA AACTGACAGA AGAGAGTGAG CACACAAAGG CACTTTGCAT

401 GTTCGATGCA TTTGCTTCTC TTGCGTGCTC ACTGCTCTAT CTGTCAGATT

451 CCGGCTCCGA TTCGGTCCCG GTCACGTTTT CTTCTTCTAA TTGTGTTCCC

501 ATCTCTCACT TTCTCTCTCA TGTGTTCTTC ATCTCTCAAA GGTAAATTAA

551 ATACGATCTG ATAATATCTG TATGAAGATT GTTCTTATTT GCTGCCATTG

601 ATTTGGTTCC CAATTGCATG CGAGTTTGCT TTTTGTATTT TCCTTTGTTG

651 AACTTTATAT TTTATGAGAT CATTAGTATC GAAAGCCTAA TCTATGAGTT

701 TAAGGCGTGG TTAAGTGAGA TTAACATGGG ACCATTAACG TTAACCTAGT

751 TTTCTTAGGC TGGTCCAAAC ACTTTTGAAT CCCTTTATTT CCTCGATCCA

801 AGTTAATATT AAATCGTTTA TCTTTTTCTT TTATCTCGTT TCTCATTAGC

851 CCTTTTAAAT CGTGATTTCT AGTCGTGATC TCTTTATAAA TATCTGTAGA

901 TATTATTTGC TCCATACGAT CATATCTATG TGTATAGTCA TGGATCTTGC

951 AAAGTACTGT TCATGATCTT TGAACTGCTA GTGATTAGTC GTTTGTTGTG

1001 CAGATTATTT ATATTGCATG CTCGTACGTT TTGATTCCAT TTATGTAATT

1051 ATGTTTCTAA TTTCGTCTAT TATATATAGT TTATTGTGTT TTGTACTACT

1101 TGTAGATGGT ATCTAATTTA TATATCATTG ATCCATGTGA TCACGTTGTG

1151 AATCTTGTGA GAATATTCTC GTGGGTTTGT TTCTTGATTT AAGAACATGC

1201 TATTTTGGTT TGTTTCTTGA TTTGAGAACA TGTTGTTTGC AGGTCCTCAA

1251 AACGACGTTT CAGACAGTGT TTGAAATTTG TTTGTTGTTT TTTTACCAAA

1301 ATCTTCAAGT GATTTGAACC TGGTAGGGAA TTTTTGGATC TAGGAAGCGA

1351 TTTGTCATTT GCAGTATCAT GATTAAGGAA TTTCCAGTAT TTGACTGCTT

1401 TATGTATGTT TCATGTCTAG ATTGTGATGA AAATTAAAAT TTGACCTTTT

1451 CTCATTTACT TTTTTTCTCT ACGCCAAAAC ACTTCAAAAT TTCATTTTTA

1501 ATTCATATAT ATTTATTGCA GGTATGAAAT GTTATCTTGG GGTTTTCTTC

1551 TGGGGGAAGA GGTGAGACAT ATTCTACAAA TCAATAAAAA CATCTAACAT

1601 TCATCGTGTC TATTGTATTG CTAGATGTCT ATTGTCATAC GTTTGTGTTG

1651 TTCTTGCTAC TGATTTGTTT CTTGCTATTG TATTGTTCCC ATGTTTTTCA

1701 TTGTTTACAA ACCGACGAAA AGAAGCCAGA TGAGATGCAA ACTTTTGTTC

1751 TTGCAATAAT TGACAACAAT TGGTTTTTAA TTTCTTCAAG AGAAATAGTA

1801 TAGTGTTTTC TTGATGCTTG TGAAATGAGT TAGCATTAAT CATTCATCGA

1851 TAATCCTCAA ACGCCATGTC TAAAAGAACG ATATGTAAGT TACAAGTTCT

1901 CTCGTTATTC TAAAGTTAGT CTGATGGAAA TGTCTCATGA TACTAAGCCA

1951 TCATTTGAAA TGGAATATAT ATGGTTTGCA GAGCTCATAT GTATACTTAG

2001 CTTGCATCAC ATTATTGCTG TGATTAAGAT GTCACTAAGG TTTTGTTTTA

2051 GAATGACATA GAGCATTTTT GGTGAGCTAG GAAGACAGAT CAAAGAGGCT

2101 CTAAGTTTGT CAAAGGTATTA GATATTTTC CGCATTTAAA TGCATTTATA

2151 ACTTGGTTGG TTTCCATATCA TAAAATTGC ATAAGTCTAG TTGTGTCTTG

2201 ATCGGAAGGC TTTTCATGTTC TGTGCAGGC TTTTGCTATT GATCACCTCT

2251 CTGATATATA TTTATTCATTC TATTTCAGG TTTGGATTAT CTATATCGAT

2301 ATTTTGGTAG CCACCCCTTTC AAATTGGTC ATGCAAAGTC AAAAAGAGAT

2351 GAATACTGCA GGGCTTTTGC GTAACCTATT TGTAATATTC TGTGGTACTG

2401 ATTAAGAGTT GATGTGCTTT GATCTATTTA ATTGATTTCG TCATGTTGCA

2451 CATATATGTT ATTGTTGTAG TTTAGAGTGT TCTGCAAGTC CACTGCAGAT

2501 TTATTATGAC AACTTGGAAT TGAAACTTGA ATCAAGAATT ATCCAAGATT

1. TAAGGAATTG ACGTAGTACC AATAGTTCAA

**Supplementary file 2:** The genomic organization of MIR156C

(At4g31877). Exons are indicated in yellow. The miR156 hairpin is underlined,

and the mature miRNA is indicated in blue. A variant 5’ end of the transcript

is indicated in green.
