# Supplementary material for: Sugar promotes vegetative phase change in Arabidopsis thaliana by repressing the expression of MIR156A and MIR156C
Source: eLife. 2013 Mar 26;2:e00260. doi: 10.7554/eLife.00260 (PMC3608266; doi:10.7554/eLife.00260)
Supplement: Supplementary file 3. — PCR primers and oligonucleotide probes. DOI: http://dx.doi.org/10.7554/eLife.00260.015 [file elife00260s003.doc]

**Supplementary file 3:** PCR primers and oligonucleotide probes

| **Primer Name** | **Sequence** |
| --- | --- |
| **For qRT-PCR** |  |
| UBQ10-R | AAAGAGATAACAGGAACGGAAACATA |
| UBQ10-F | GGCCTTGTATAATCCCTGATGAATAA |
| TUB2-F | AGCAATACCAAGATGCAACTGCG |
| TUB2-R | TAACTAAATTATTCTCAGTACTCTTCC |
| qSPL3-F | CTTAGCTGGACACAACGAGAGAAGGC |
| qSPL3-R | GAGAAACAGACAGAGACACAGAGGA |
| qSPL9-F | CAAGGTTCAGTTGGTGGAGGA |
| qSPL9-R | TGAAGAAGCTCGCCATGTATTG |
| qSPL13-F | CCAATCTCTTCTTCTCCAAACAGTACCAGAAGC |
| qSPL13-R | GAAGCAAATGAGGGACTGACGACG |
| qMIR156A-F | CTTCGTTCTCTATGTCTCAATCTCTC |
| qMIR156A-R | TGATTAAAGGCTAAAGGTCTCCTC |
| qMIR156B-F | GCTAGAAGAGGGAGAGATGGTGATTGAG |
| qMIR156B-R | GTGAGCACGCACACGCAAAGTTATAGAC |
| qMIR156C-F | GTGATAATGAGTGATGACTGATG |
| qMIR156C-R | GAAAACGTGACCGGGACCGAATCG |
| qMIR156D-F | GGGAAGTTGTATAAAAGTTTTGTATATGG |
| qMIR156D-R | TGGTATGCAGAGACAGATAAGAAC |
| qMIR156F-F | GATGAAGCAAGTCAACTAAAGGAG |
| qMIR156F-R | GCAGGAGACAAGAAGAGAGTAAG |
| qMIR156H-F | GAAAGAGAGCACAACCTGGGATTAGC |
| qMIR156H-R | CGCAATGATGGTGGCAGAAGGAAAGAG |
| GUS plus-F | CGTCCAAGGAAACAAGAAGGG |
| GUS plus-R | AGCGTTCTTGTAGCCGAAATC |
|  |  |
| **For MIR156A reporter** |  |
| P156a-5'-F1(EcoR I) | GAATTCGTTTGAGAATGTGTCTTGTAAAAGTGACAGATCC |
| P156a-5'-R1(Nco I) | CCATGGGTTTCTTTGCGTTTCTCTTGTCCC |
| P156a-3'-F1(Pml I) | CACGTGGATTCCGGTGCTGATCTCTTTGGCC |
| p156a-3'-R1(BstE II) | GGTGACCGTTGTCTACTTTGTTTGATATGTGACGAC |
|  |  |
| **For MIR156C reporter** |  |
| P156c-5’-F (NcoI) | CCATGGGTTTCTATGCGTTTCTCTTAAAATTTGTCCC |
| P156c-5’-R(XmaI) | CCCGGGGGTGGAAAGCGATAAGCGTGTGG |
| P156c-3’-F(BstE II) | GGTGACCCTGCAAAAGTTCTTTGTAAGACTTTATATACAC |
| P156c-3’-R(BstE II) | GGTGACCGATTCCGGCTCCGATTCGGTCCCGG |
|  |  |
| **For miRNA blot** |  |
| miR156 | GTGCTCACTCTCTTCTGTCA |
| U6 | AGGGGCCATGCTAATCTTCTC |
| tRNA-met | TCGAACTCTCGACCTCAGGAT |
|  |  |
| **For genotyping** |  |
| SALK_056809-RP | AAAGAGATCAGCACCGGAATC |
| SALK_056809-LP | CGCGCTTCACTTAAAATTACG |
| SALK_131562-RP | CGCGCTTCACTTAAAATTACG |
| SALK_131562-LP | AAAGAGATCAGCACCGGAATC |
| GT22288-RP | AAAGAGATCACGACTAGAAATCACG |
| GT22288-LP | AACAGATTCTTCCTCTCTTCTCC |
